# Essential and non-essential DNA replication genes in the model halophilic Archaeon, *Halobacterium *sp. NRC-1

**DOI:** 10.1186/1471-2156-8-31

**Published:** 2007-06-08

**Authors:** Brian R Berquist, Priya DasSarma, Shiladitya DasSarma

**Affiliations:** 1 University of Maryland Biotechnology Institute, Center of Marine Biotechnology, Baltimore, Maryland 21202 USA

## Abstract

**Background:**

Information transfer systems in Archaea, including many components of the DNA replication machinery, are similar to those found in eukaryotes. Functional assignments of archaeal DNA replication genes have been primarily based upon sequence homology and biochemical studies of replisome components, but few genetic studies have been conducted thus far. We have developed a tractable genetic system for knockout analysis of genes in the model halophilic archaeon, *Halobacterium *sp. NRC-1, and used it to determine which DNA replication genes are essential.

**Results:**

Using a directed in-frame gene knockout method in *Halobacterium *sp. NRC-1, we examined nineteen genes predicted to be involved in DNA replication. Preliminary bioinformatic analysis of the large haloarchaeal Orc/Cdc6 family, related to eukaryotic Orc1 and Cdc6, showed five distinct clades of Orc/Cdc6 proteins conserved in all sequenced haloarchaea. Of ten *orc*/*cdc6 *genes in *Halobacterium *sp. NRC-1, only two were found to be essential, *orc10*, on the large chromosome, and *orc2*, on the minichromosome, pNRC200. Of the three replicative-type DNA polymerase genes, two were essential: the chromosomally encoded B family, *polB1*, and the chromosomally encoded euryarchaeal-specific D family, *polD1/D2 *(formerly called *polA1/polA2 *in the *Halobacterium *sp. NRC-1 genome sequence). The pNRC200-encoded B family polymerase, *polB2*, was non-essential. Accessory genes for DNA replication initiation and elongation factors, including the putative replicative helicase, *mcm*, the eukaryotic-type DNA primase, *pri1/pri2*, the DNA polymerase sliding clamp, *pcn*, and the flap endonuclease, *rad2*, were all essential. Targeted genes were classified as non-essential if knockouts were obtained and essential based on statistical analysis and/or by demonstrating the inability to isolate chromosomal knockouts except in the presence of a complementing plasmid copy of the gene.

**Conclusion:**

The results showed that ten out of nineteen eukaryotic-type DNA replication genes are essential for *Halobacterium *sp. NRC-1, consistent with their requirement for DNA replication. The essential genes code for two of ten Orc/Cdc6 proteins, two out of three DNA polymerases, the MCM helicase, two DNA primase subunits, the DNA polymerase sliding clamp, and the flap endonuclease.

## Background

Archaeal microorganisms, though prokaryotic, are phylogenetically distinct from bacteria [1] and exhibit considerable similarities to eukaryotes in their macromolecular biosynthetic machinery, particularly with respect to their DNA replication system. Among the Archaea, *Halobacterium *sp. NRC-1 provides an excellent model system to address questions of fundamental DNA replication biology using bioinformatic, genomic, and genetic approaches [2]. The genome is relatively small, comprised of a 2 Mbp large chromosome and two minichromosomes, pNRC200 (365 kbp) and pNRC100 (191 kbp), and codes 2,682 putative genes. Of these, only 2,532 genes are unique, due to duplication of 145,428 bp between the two extrachromosomal replicons [3]. *Halobacterium *sp. NRC-1 is easily cultured in the laboratory in hypersaline media containing 4.3 M NaCl and has well-developed genetic methodology, including a facile transformation system, plasmid shuttle vectors, selectable markers, and a directed gene knockout/replacement system [4,5].

**Figure 1:**
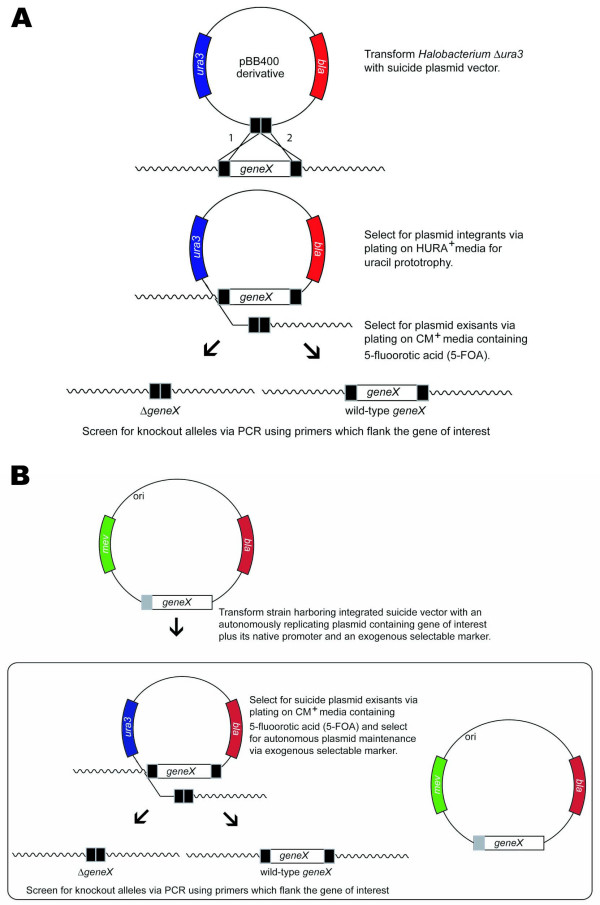
A. Gene knockout strategy in *Halobacterium *sp. NRC-1. In this approach, a targeted gene allele, shown here as a deletion, is first cloned into the suicide plasmid, pBB400, which is capable of replication in *E. coli *(but not in *Halobacterium*). The plasmid also contains the native *ura*3 gene under the control of its own promoter. The resulting plasmid is introduced into a *Halobacterium *sp. NRC-1Δ*ura*3 host via transformation. Integrants are then selected by uracil prototrophy (Ura^+^) using commercially available uracil-dropout media components (HURA^+ ^media). Subsequently, plasmid excisants are selected via counterselection of *ura*3, 5-Foa-resistance (Foa^r^). This gives rise to derivatives containing either the original or mutant allele, which may be distinguishable by PCR or phenotypic analysis. B. A method for construction of chromosomal knockouts of essential genes. A complementation strategy is shown where an autonomously replicating plasmid vector which contains a functional gene of interest, *geneX*, is introduced into the host strain, e.g. by selection for mevinolin resistance (Mev^r^). Strains containing a knockout of the chromosomal copy may then be selected using the method described in part a, with the additional selection for the complementing plasmid with Mev^r^.

For gene knockouts in the *Halobacterium *sp. NRC-1 system, we developed a method employing the selectable and counterselectable *ura3 *gene (Fig. 1) [6,7]. The system also utilizes a suicide plasmid vector with two essential elements, a wild-type copy of the *Halobacterium *sp. NRC-1 *ura3 *gene plus its native promoter, and at least 500 bp of 5' and 3' DNA flanking the targeted gene. Transformation of an isogenic *Halobacterium *sp. NRC-1 strain containing a deletion of the chromosomal *ura3 *gene with the suicide vector, followed by selection for uracil prototrophy results in an integrated copy of the suicide vector at the genomic locus homologous to the targeted gene. Counterselection for suicide vector loss is accomplished by selection for 5-fluoroorotic acid (Foa) resistance and colonies are then screened via polymerase chain reaction (PCR) to discriminate between knockout and wild-type alleles. Excision of the suicide plasmid vector can occur on the same side as the integration, yielding restoration of the wild-type allele, or excision can occur on the opposite side of the integration, yielding replacement of the wild-type gene with a deletion of the targeted gene. In cases of essential genes, a functional copy of the targeted gene must be provided on a replicating plasmid to recover deletants. This gene knockout system has been successfully employed for studies of several gene clusters in *Halobacterium *sp. NRC-1 [2,5].

In addition to the gene knockout system, a genetic screen for the isolation of autonomously replicating sequences (ARS) was established for *Halobacterium *sp. NRC-1. Earlier genetic work identified two likely replication origins in *Halobacterium *sp. NRC-1 via cloning of ARS elements, one on the large chromosome, and another located within the common region of pNRC100 and pNRC200 [8,9]. Sequence analysis of the pNRC minimal replicon showed the requirement of a unique gene, *repH*, and an AT rich region 5' to the gene. Mutation or deletion of either the AT rich sequence or the *repH *gene was found to abolish autonomous replication ability of plasmids [9]. For the large chromosome, the ARS element was found directly 5' to *orc*7, one of ten *orc*/*cdc6 *genes in the genome, in a region of GC skew polarity switch [10] and global minimum in Z curve analyses [11]. However, regions proximal to two other chromosomal *orc*/*cdc6 *genes, *orc6 *and *orc8*, could not confer autonomous replication ability. The chromosomal ARS region contained unusual sequence elements: a large (33 bp) inverted repeat flanking an AT rich region of 189 bp plus the *orc7 *gene. Genetic analysis showed that both the inverted repeats, the AT rich region, as well as the *orc7 *gene were required for autonomous replication ability [8]. Work in other archaeal organisms identified chromosomal DNA replication origin(s) comprised of similar sequence elements proximal to *orc7 *homologs in the genomes of *Pyrococcus abyssi *[12-14] and *Sulfolobus *spp. [15,16].

**Figure 2:**
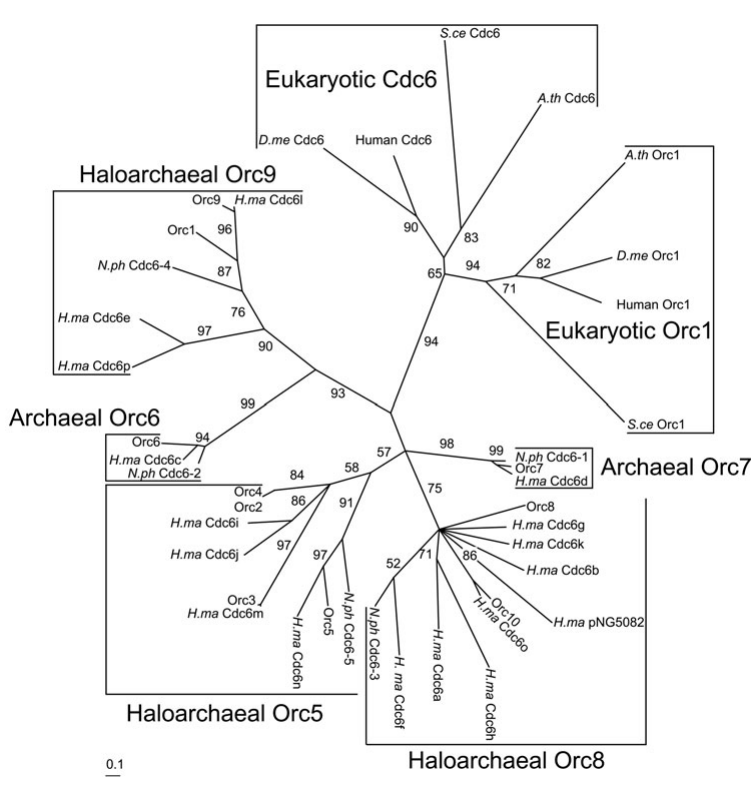
Quartet puzzling consensus maximum likelihood phylogenetic tree of Orc1 and Cdc6 protein sequences from representative eukaryotes and Orc/Cdc6 protein sequences from published haloarchaeal genome sequences. Protein sequences from *Halobacterium *sp. NRC-1 are denoted as their published protein names. Sequences from *H. marismortui *(*H.ma*), *N. pharaonis *(*N. ph*), *Saccharomyces cerevisiae *(*S.ce*), *Drosophila melanogaster *(*D.me*), and *Arabidopsis thaliana *(*A.th*) use three letter designations and published protein names. *Homo sapiens *sequences are denoted as Human along with their published protein names.

In addition to genetic studies, predicted replisome components of haloarchaea have been identified via bioinformatic analysis [17]. One of the most interesting findings was the presence of a large family of *orc*/*cdc6 *genes in *Halobacterium *sp. NRC-1 and other haloarchaea, homologous to eukaryotic origin recognition complex (ORC) proteins 1, 4, and 5 as well as to the eukaryotic replicative helicase loader Cdc6 (Fig. 2) [8,18]. This finding suggested that multiple Orc proteins in *Halobacterium *sp. NRC-1 may be required for replication, perhaps through formation of heteromeric protein complexes for origin recognition. Many additional genes coding eukaryotic-type DNA replisome components have also been found, with homology to replicative helicase proteins (MCM), ssDNA binding proteins (RFA), processivity clamp loader proteins (RFC), processivity clamp protein (PCNA), primase proteins, Okazaki fragment maturation proteins (Rad2 and RNaseH), ATP dependant DNA ligase, DNA polymerases (B family), and type IIB topoisomerase (Top6A and B). The novel heterodimeric family D DNA polymerase found only in the euryarchaea is also present in *Halobacterium *sp. NRC-1 [19]. A few genes for bacterial-type replication proteins, e.g. a primase (DnaG), and type IA (TopA) and IIA DNA topoisomerases (GyrA and B), are also present [17].

With the availability of an inventory of replication factors likely acting at haloarchaeal DNA replication origins and a facile gene knockout system, we sought to answer basic questions regarding the essentiality of DNA replication gene assignments in an archaeon. The inability to recover deletion mutants indicates the requirement of genes coding two Orc/Cdc6 proteins, two different replicative DNA polymerases, a replicative helicase, a eukaryotic-type primase, a DNA polymerase sliding clamp, and the flap endonuclease. Eight of the *orc/cdc6 *genes and a *polB *gene are dispensable to cells. This study shows the first *in vivo *evidence for genes likely to be critical for DNA replication in Archaea.

## Results

### Bioinformatic analysis of Orc/Cdc6

Halophiles are unique among the Archaea in possessing a large gene family of Orc/Cdc6 genes, as other archaeal organisms most commonly encode only two Orc/Cdc6 homologs [8]. In *Halobacterium *sp. NRC-1, ten *orc/cdc6 *genes are present, with *orc6*, *orc7*, *orc8*, and *orc10 *genes located on the large chromosome, *orc1*-*5 *located on pNRC200, and *orc9 *located on both the pNRC100 and pNRC200 replicons. The gene products are quite diverse, ranging from 21–91% similarity (data not shown), with Orc2 and Orc4 being the most similar overall and Orc8 and Orc10 being the most similar encoded chromosomally. The haloalkaliphilic archaeon, *Natronomonas pharaonis*, encodes the fewest number of *orc*/*cdc6 *genes among haloarchaea (five), while *Haloarcula marismortui *encodes the most (seventeen). Phylogenetic reconstruction of Orc/Cdc6 protein sequences from sequenced haloarchaeal genomes and representative eukaryotes indicated the presence of five distinct haloarchaeal/archaeal clades, all distantly related to eukaryotic Orc1 and Cdc6 (Fig. 2). The general archaeal clades, Orc6 and Orc7, have just single members from each haloarchaeon, while all other haloarchaeal-specific clades have multiple members from *Halobacterium *sp. NRC-1 and *H. marismortui*, and a single member from *N. pharaonis *(Fig. 2).

### Knockout of *orc/cdc6 *Genes

One of our primary goals was to determine how many and which of the *orc/cdc6 *genes in the *Halobacterium *sp. NRC-1 genome are essential (Table 1). Using a directed gene knockout approach all ten *orc *genes were individually targeted for in-frame deletion. To this end, we constructed suicide plasmids containing at least 500 bp of 5' and 3' flanking DNA sequences of all ten *orc *genes (designed to leave only 5–13 codons after deletion, see Table 1) and introduced them into a Δ*ura3 *derivative of *Halobacterium *sp. NRC-1. Excision of the integrated suicide plasmid may occur on the same side as integration (yielding restoration of the wild-type allele), or on the opposite side as the integration (yielding replacement of the wild-type gene with the deletion allele). In theory, for a nonessential gene, either event should be recovered with the same frequency, yielding 50% wild-type restoration and 50% deletion allele replacement (Fig. 1A). In contrast, for essential genes, loss of the wild-type gene allele would results in loss of viability, so only the wild-type recombinant would be recovered.

**Table 1:** Construction of gene knockout and complementation plasmids.

Plasmid name	Primer position	Primer Sequence 5'-3'	Number of 5' codons	Number of 3' codons
pBBΔ*orc1*	5' forward	CGCAAGCTTGACTCCACCCTTCCGAGAGT	3	2
	5' reverse	CGCACTAGTGGTGATCATGGGTTTGCGTC		
	3' forward	CGCACTAGTCGAAACTAGCTCTCCAAGCTC		
	3' reverse	CGCGGATCCTCTACTGTACAGCAGATGAG		
pBBΔ*orc2*	5' forward	CGCAAGCTTCAACAAAATTATGCGTAGAG	2	3
	5' reverse	CGCACTAGTCGTCATTGAATATCACACGG		
	3' forward	CGCACTAGTGCCACGTTCTGAGTATTCTG		
	3' reverse	CGCGGATCCGATCTTGTACTTGTCCTCGC		
pBBΔ*orc3*	5' forward	CGCGGATCCTGAAACGGGTCTGTGAGTGG	4	6
	5' reverse	CGCACTAGTCCCTTTCACCATCTCGATAA		
	3' forward	CGCACTAGTCAGACGTTGATGGGATGGTGA		
	3' reverse	CGCGAATTCGGTGAGGACCTCGAGTTCGAT		
pBBΔ*orc4*	5' forward	CGCAAGCTTCAGGAGACAGCTACCTACGA	3	2
	5' reverse	CGCACTAGTGGGCGTCATTGAATATCACA		
	3' forward	CGCACTAGTCGCGAGTAATGACCCCTACT		
	3' reverse	CGCGGATCCTCGGCTATCAAGGGTTCAGC		
pBBΔ*orc5*	5' forward	CGCAAGCTTAGTTGGTGACGCTCATCGGC	4	2
	5' reverse	CGCGAATTCGCGACCAGCCATACGTATGC		
	3' forward	CGCGAATTCGAGGAGTAGTTGCCAGGCGA		
	3' reverse	CGCGGATCCTGATCGAGGCGAACACGCTG		
pBBΔ*orc6*	5' forward	CCGGAATTCTTCGAGGCGACGCTGCGGGA	7	6
	5' reverse	CTCTTCGGGGTCCTCATCCAT		
	3' forward	GCGGTCCTGGAGCGCCTGTAG		
	3' reverse	CCGGAATTCCAGCGCCTCAACCCGATCGAC		
pBBΔ*orc7*	5' forward	CGCGGATCCGCGCCCGAACGCAACTAGAA	3	3
	5' reverse	GCGCTCGAGGTCTGTCATGTATTCACGCAC		
	3' forward	GCGCTCGAGGAGAACAATTAGTGGATGCT		
	3' reverse	GCGAAGCTTGGTGTGATGTTCATGACCAT		
pBBΔ*orc8*	5' forward	CCGGAATTCCACGTCGTGTTGGCGGTGGT	3	3
	5' reverse	GCGCTCGAGCGTCTTCATCGCTTGCCGAGA		
	3' forward	GCGCTCGAGGCGACCGTGTAGACCCCGGA		
	3' reverse	CGCAAGCTTGATGAAGCTCCGCCGCAGCG		
pBBΔ*orc9*	5' forward	CGCAAGCTTTGGGTCGTGTACACGGCCTC	4	3
	5' reverse	CGCACTAGTGCGGCAGGTCATATAAGAGT		
	3' forward	CGCACTAGTGTAACCCAATAAGCTGCGAA		
	3' reverse	CGCGGATCCAACACTCATCGACGAGTGAA		
pBBΔ*orc10*	5' forward	CCGGATCCCCTGTGGTCGTTCTGGAAGAC	5	2
	5' reverse	CCACTAGTACCCAACAGTGACATCCTCC		
	3' forward	CGCACTAGTGTGCTGTAGCGATTGTGCGA		
	3' reverse	CGCCTCGAGCACGGAGACGTCGAGAGCGAG		
pBBΔ*polD1*	5' forward	CCGGAATTCGTCGGTCGGATCGGGGACAT	2	2
	5' reverse	GCGCTCGAGCGTCATGTCCCCGTCGATCT		
	3' forward	GCGCTCGAGTTCTCGTAGCCACGGCGGCG		
	3' reverse	GCGAAGCTTCGTTGTGCACCATCGTCACC		
pBBΔ*polD2*	5' forward	GCGGAATTCGGCGTGGCGGTAACGGCGTT	1	2
	5' reverse	GCGACTAGTCATTACAGCCAGCGGTCGAG		
	3' forward	GCGACTAGTTTCATGTGACCACGGCGCTC		
	3' reverse	GCGAAGCTTGACGTGATCGACGAACACACC		
pBBΔ*polB1*	5' forward	CCGGAATTCGCCAACACTGCCGCGTTGAA	3	3
	5' reverse	CGCACTAGTGTTTCCCATTGGGTTCGGGT		
	3' forward	CGCACTAGTCAGTTCACGTAGCCCGCTGG		
	3' reverse	CGCAAGCTTCTCGTCAACAACGCCGGGCT		
pBBΔ*polB2*	5' forward	CGCGAATTCCCGAACGACGCGGCATCAAG	2	2
	5' reverse	CGCACTAGTCGGCATTCCCTACAGAACCA		
	3' forward	CGCACTAGTCTGTCGTAGTGGACCCCACC		
	3' reverse	CGCAAGCTTCGGCTTTTCCTGGCCAAGTC		
pBBΔ*mcm*	5' forward	CGCGAATTCGTCGAGAACCCCAGGATGAG	3	3
	5' reverse	CGCACTAGTCGGATCCATCTGGTAGAGAT		
	3' forward	CGCACTAGTCGCTCGATCTAGCCGACGGC		
	3' reverse	CGCAAGCTTACAGCACGCCCACGTGCTCGT		
pBBΔ*pri1*	5' forward	ATGAGTCCCCCACTCGGTCTT	2	6
	5' reverse	GTGCATGCCGGCAATCGTGG		
	3' forward	GCCGAGAAGGCCACAGAATGA		
	3' reverse	GGCGGCTCTTCGACCTGACT		
pBBΔ*pri2*	5' forward	TCTGTCAGGACCGGGCCACT	2	2
	5' reverse	GTTCATGCTGGCCCGTGTTTG		
	3' forward	CCCGATTAGCCCTGCTTGCC		
	3' reverse	ATGGCTAACTCCAACGCCAA		
pBBΔ*pcn*	5' forward	TCTCGTCTGCGGCGGGGGTA	4	4
	5' reverse	CGCCTTGAACATTATTGCAGA		
	3' forward	ATCCAGTCCAACTGACGCCA		
	3' reverse	ATGCTGGCCCGTGTTTGCGA		
pBBΔ*rad2*	5' forward	CCGGAATTCGTGATGTCGAACACGGGGAA	3	2
	5' reverse	CGCCTCGAGGTTCCCCATCACGCCCAGTT		
	3' forward	CGCCTCGAGTGGACGTGACCGGCGCTGAT		
	3' reverse	CGCAAGCTTGCGTAAAAGCCATCGGAACC		
pBB*polD1*all	5' forward	CGCGAATTCCATACCGGTTCGGTCGTACC		
	3' reverse	CGCACTAGTCAGGAAGTCCTCTCGCATAC		
pBB*polB1*all	5' forward	CGCGAATTCGTCACCTCGGTCCGTGGTAG		
	3' reverse	CGCACTAGTGCGTTCGCGGCGACCCAGAGT		
pBB*mcm*all	5' forward	CGCGAATTCGAACAGCATGAACATGCCGA		
	3' reverse	CGCACTAGTCGAATACGCCACTGCTAACAA		
pBB*pri2*all	5' forward	CGCGAATTCGGCGTACTTCCACGTCCAGGG		
	3' reverse	CGCACTAGTGCCACGTCCGGGTACGCAGTG		
pBB*rad2*all	5' forward	CGCGAATTCCCAGCACGAGTCGAGTGGTAA		
	3' reverse	CGCACTAGTATCCATGCCTGTGCGTGAGC		

**Figure 3:**
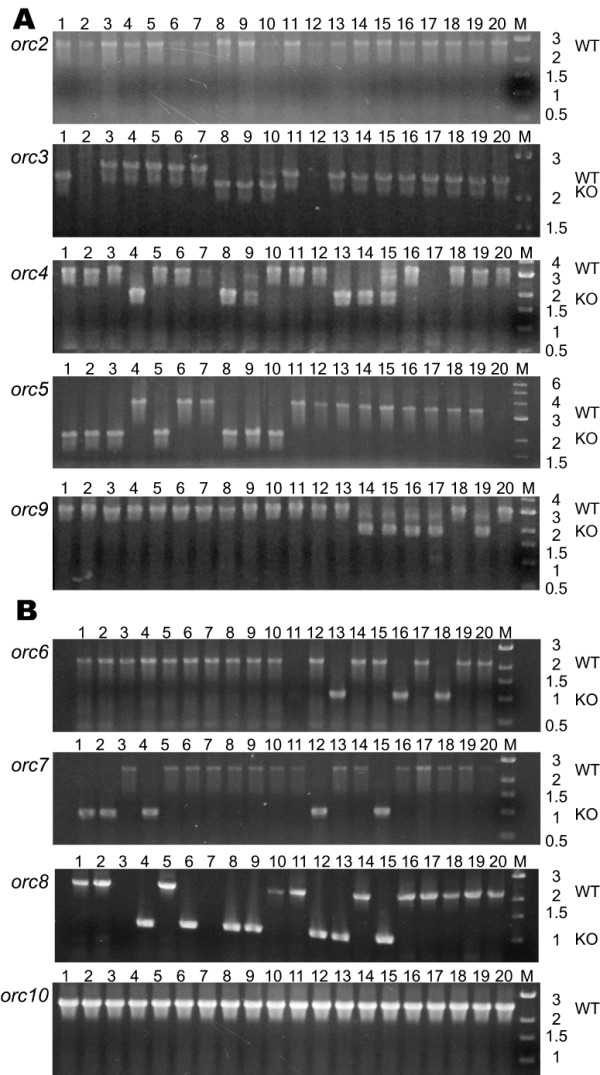
PCR assay to screen for knockout alleles of *Halobacterium *sp. NRC-1 *orc *genes. Lanes 1–20 contain products obtained from individual PCR reactions using total genomic DNA extracted from 20 individual Foa^r ^colonies as template for each gene examined respectively, M denotes DNA ladder. A. Extrachromosomal *orc *genes. Primers residing ~1000 bp 5' and 1000 bp 3' to each *orc *gene (*orc2*, *orc3*, *orc4*, *orc5*, or *orc9*) in *Halobacterium *sp. NRC-1 were used with total genomic DNA from individual colony isolates in PCR reactions to screen for *orc *gene knockouts. For *orc2*, *orc3*, *orc4*, *orc5*, and *orc9*, knockout alleles where obtained are ~2000 bp in size, while wild-type alleles are approximately 800, 300, 1200, 1400, and 1000 bp larger, respectively. B. Chromosomal *orc *genes. Primers residing ~500 bp 5' and ~500 bp 3' to each chromosomally encoded *orc *gene (*orc6*, *orc7*, *orc8*, *orc10*) in *Halobacterium *sp. NRC-1 were used with total genomic DNA from individual colony isolates in PCR reactions to screen for *orc *gene knockouts. For *orc6*, *orc7*, *orc8*, and *orc10*, knockout alleles have a size of ~1000 bp where obtained, wild-type alleles are approximately 1100, 1500, 1200, and 1400 bp larger, respectively.

Based upon the requirement of *orc7 *for minichromosome plasmid replicon autonomous replication and *orc6 *conservation in the genome sequences of other Archaea [8], we expected that these two *orc *genes would likely be essential for normal growth. Surprisingly, we found that neither *orc7 *nor *orc6 *were essential, nor were *orc3*, *orc4*, *orc5*, *orc8*, or *orc9 *(Fig. 3A and 3B), since knockouts were readily obtained for those genes (Table 2). [Interestingly, during the process of screening for *orc1 *knockout strains it was observed that a natural event in the host strain deleted *orc1*, indicating non-essentiality for *orc1 *(data not shown).] In all these cases, between 15 and 30 % of Foa^r ^isolates of integrants were knockouts. In contrast, however, we did not find any deletions of two *orc *genes, *orc10*, located on the large chromosome, and *orc2*, present on pNRC200, indicating that these genes are essential, which is consistent with their involvement in DNA replication. Orc10 belongs to a clade of uniquely haloarchaeal Orc proteins along with Orc8, eight other Orc/Cdc6 members from the distantly related archaeon *H. marismortui*, and a single member from *N. pharaonis *(Fig. 2). Orc2 also belongs to part of a larger haloarchaeal clade of Orc/Cdc6 homologs that includes Orc3, Orc4, Orc5, four additional members from *H. marismortui*, and one member from *N. pharaonis *(Fig. 2).

**Table 2:** Statistics for replication gene knockouts in *Halobacterium *sp. NRC-1.

	Gene Knockout				Complementation	
		
Gene name	# Colonies screened	# KO obtained	% KO obtained	P-value	# Colonies screened	# KO obtained
*orc2*	40	0	0	1.01(10)^-5^		
*orc3*	20	4	20	N.A.		
*orc4*	20	6	30	N.A.		
*orc5*	20	7	35	N.A.		
*orc6*	20	4	20	N.A.		
*orc7*	20	6	30	N.A.		
*orc8*	40	6	15	N.A.		
*orc9*	20	5	25	N.A.		
*orc10*	80	0	0	1.01(10)^-10^		
*polD1*	40	0	0	1.01(10)^-5^	20	6
*polD2*	40	0	0	1.01(10)^-5^		
*polB1*	40	0	0	1.01(10)^-5^	15	1
*polB2*	20	5	25	N.A.		
*mcm*	40	0	0	1.01(10)^-5^	20	15
*pri1*	40	0	0	1.01(10)^-5^		
*pri2*	40	0	0	1.01(10)^-5^	20	1
*pcn*	40	0	0	1.01(10)^-5^		
*rad2*	40	0	0	1.01(10)^-5^	20	9

N.A – Not Applicable

### Two replicative-type DNA polymerases are essential in euryarchaea

**Figure 4:**
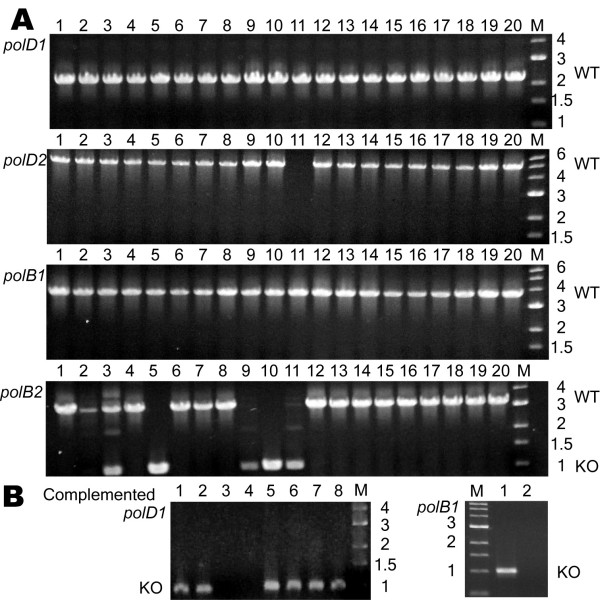
PCR assay to screen for knockout alleles of DNA polymerase genes *polD1*, *polD2*, *polB1*, and *polB2 *in *Halobacterium *sp. NRC-1. A. For the top four panels, lanes 1–20 contain product obtained from individual PCR reactions using total genomic DNA extracted from 20 individual 5-Foa^r ^colonies as template and primers which reside ~500 bp 5' and 500 bp 3' of the specific ORF targeted for deletion. For *polD1*, *polD2*, *polB1*, and *polB2*, knockout alleles are ~1000 bp in size where obtained, while wild type alleles are approximately 1200, 4100, 2700, and 2200 bp larger, respectively. B. The two panels at the bottom show the same screens as above, but using *Halobacterium *sp. NRC-1 derivatives with a replicating plasmid containing a wild-type copy of the *polD1 *or *polB1 *gene plus the entire 5' intergenic region. Only the ~1,000 bp knockout alleles are observed.

All euryarchaeal genomes encode DNA polymerases belonging to two different families (B and D) [20]. In *Halobacterium *sp. NRC-1, four DNA polymerase genes were targeted for individual deletion: *polD1 *and *polD2*, the chromosomally encoded small and large subunits of the heterodimeric euryarchaeal specific D family DNA polymerase, and *polB1 *and *polB2*, two genes encoding separate B family DNA polymerases, one on the large chromosome and one on pNRC200. In each case, suicide plasmids containing ~500 bp 5' and 3' to the genes (including 3–6 codons; Table 1) were constructed and integrants were selected by uracil prototrophy. After isolation and screening of 40 Foa^r ^colonies via PCR, we found that deletion alleles could not be recovered for either gene of the D family DNA polymerase, *polD1 *and *polD2*, or for the gene encoding the chromosomally encoded B family polymerase, *polB1*, indicating that they are essential to this organism (Fig. 4A and Table 2). In contrast, deletions of the second B family DNA polymerase gene, *polB2*, encoded on pNRC200, were readily obtained (25 % of Foa^r ^colonies), indicating that this gene is dispensable to the cell (Fig. 4A).

### Archaeal *mcm *is an essential gene

**Figure 5:**
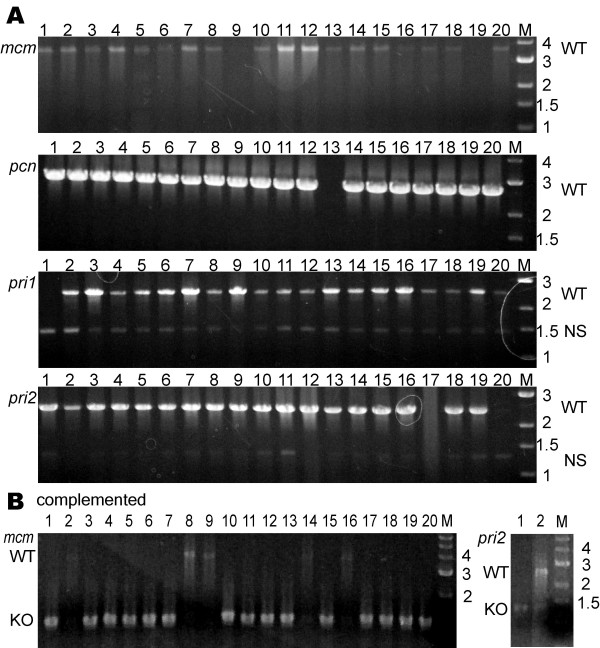
PCR assay to screen for knockout alleles of *mcm*, *pcn, pri1*, and *pri2 *in *Halobacterium *sp. NRC-1. A. For the top four panels, lanes 1–20 contain product obtained from individual PCR reactions using total genomic DNA extracted from 20 individual Foa^r ^colonies as template and primers which reside ~500 bp 5' and 500 bp 3' to either *mcm*, *pri1*, and *pri2 *or ~1000 bp 5' and ~1000 bp 3' to *pcn*. For *mcm*, *pri1*, and *pri2*, predicted knockout alleles would be ~1000 bp in size, while wild-type alleles are approximately 2500, 1300, and 1100 bp larger, respectively. For *pcn *predicted knockout alleles would be ~2000 bp in size, while wild-type alleles are approximately 800 bp larger. For *pri1 *and *pri2 *NS refers to a nonspecific PCR based artifact that is observed when using those specific primer sets. B. The two panels at the bottom show the same screens as above, but after using *Halobacterium *sp. NRC-1 derivatives with a replicating plasmid containing a wild-type copy of the *mcm *or *pri2 *gene plus the entire 5' intergenic region. Either the ~1,000 bp knockout alleles or larger wild-type alleles are observed.

MCM is an essential complex for DNA replication in eukaryotes and is the likely replicative DNA helicase. To investigate whether *mcm *is required in Archaea, we targeted the single *mcm *gene in *Halobacterium *sp. NRC-1 for deletion. A suicide plasmid containing ~500 bp flanking the *mcm *gene (including 6 codons; Table 1) was constructed and integrants were selected by uracil prototrophy. Screening of 40 Foa^r ^colonies via PCR, resulted in no recovery of deletants of the *mcm *gene (Fig. 5A and Table 2), even though *Halobacterium *sp. NRC-1 possesses genes for over a dozen other predicted DNA/RNA helicases [17], displaying that this gene is essential.

### Genes of the eukaryotic-type DNA dependent RNA primase are essential in Archaea

In order to address whether the eukaryotic-type primase was essential, directed in-frame deletions of *Halobacterium *sp. NRC-1 *pri1 *and *pri2 *genes were attempted. Once again, suicide plasmids containing ~500 bp 5' and 3' to the genes were constructed (including 8 or 4 codons, respectively, Table 1) and integrants were selected by uracil prototrophy. After isolation and screening 40 Foa^r ^colonies of each integrant, we observed no deletants for either *pri1 *or *pri2 *providing *in vivo *data supporting the requirement of eukaryotic-type primases in Archaea (Fig. 5A, Table 2).

### Archaeal PCNA is essential

The gene for PCNA is known to be essential in eukaryotes, so we wanted to determine whether *pcn *is essential in Archaea as well. Utilizing the *ura3 *based targeted gene knockout system in *Halobacterium *sp. NRC-1, a suicide plasmid containing ~500 bp 5' and 3' to the *pcn *gene, including an in-frame deletion with 8 codons (Table 1), was constructed and integrants were selected by uracil prototrophy. After isolation and screening 40 Foa^r ^colonies, we were unable to observe any deletants of *pcn*, indicating that this gene is indeed essential (Fig. 5A, Table 2).

### The Rad2 family flap endonuclease is essential in Archaea

**Figure 6:**
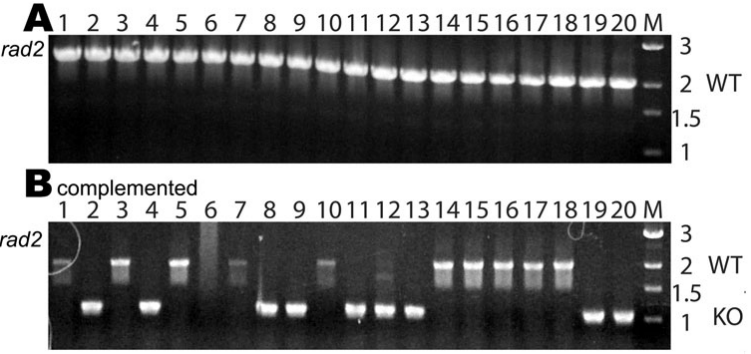
Implementation of complementation strategy for *rad2*. For each, Lanes 1–20 contain product obtained from individual PCR reactions using total genomic DNA extracted from 20 individual Foa^r ^colonies as template and primers which reside ~500 bp 5' and 500 bp 3' of the *rad2 *gene targeted for deletion. A. PCR assay to screen for knockout alleles of flap endonuclease *rad2 *gene in *Halobacterium *sp. NRC-1. B. PCR assay to screen for knockout alleles of flap endonuclease *rad2 *gene in *Halobacterium *sp. NRC-1 derivatives transformed with a replicating plasmid containing a wild-type copy of the *rad2 *gene plus the entire 5' intergenic region. Both the wild-type and deletion alleles are observed.

In order to determine whether the *Halobacterium *sp. NRC-1 *rad2 *gene likely coding for the putative flap endonuclease was essential, the gene was targeted for deletion via our *ura3 *based knockout system. A suicide plasmid vector which contained ~500 bp 5' and 3' to the *rad2 *gene, including an in-frame deletion containing 5 codons was constructed (Table 1). After isolation and screening 40 Foa^r ^colonies, we were unable to recover any deletants of *rad2 *(Fig. 6A), indicating that this gene is essential.

### Statistical analysis of DNA replication gene knockouts

In our knockout experiments, we observed the average frequency for wild-type restoration to be ~75% and the frequency for deletion allele replacement to be ~25% for non-essential genes regardless of the genomic locus [6,7,21-23]. To determine the confidence with which we could conclude the essentiality of genes for which we did not obtain knockouts, we tested for rejection of the null hypothesis. For a typical case, where H_0_=*geneX *is non-essential, and the probability of identifying the wild type allele, P_WT_, is 0.75, by screening 40 Foa^r ^colonies, the probability of finding 100% wild-type restoration is calculated to be 10^-5^, if the gene is non-essential. In other words, there is a 1 in 100,000 chance that a gene knockout would not be obtained if the gene was non-essential, providing a confidence level of greater than 99.999 % probability of identifying a knockout of a non-essential gene when screening through 40 individual Foa^r ^colonies. Therefore, very strong evidence is provided to reject the null hypothesis that the gene is non-essential, indicating that the target gene is indeed essential.

All target genes where no deletion was obtained were tested for rejection of the null hypothesis. A minimum of 40 and a maximum of 80 colonies were screened in each case. For the *orc2*, *mcm, polD1, polD2, polB1, pri1, pri2, pcn*, and *rad2 *genes, 40 Foa^r ^colonies were screened without recovering a single knockout, indicating that the probability of these genes being essential is > 99.999 % (*i.e*. less than a 1 in 100,000 chance that these genes are non-essential). For *orc10*, 80 Foa^r ^colonies were screened without identifying a single knockout, indicating a probability > 99.9999999 % of this gene being essential (*i.e*. less than a 1 in 10,000,000,000 chance that this gene is non-essential) (Table 2).

### Complementation and knockout analysis of essential DNA replication genes

To further validate the strong statistical evidence supporting the essential nature of some DNA replication genes, we performed knockout analysis in the presence of a complementing gene for a select subset of the essential genes. This complementation analysis involved placing a wild-type copy of the gene of interest, plus its native promoter, on a plasmid capable of replication and the selectable mevinolin-resistance (Mev^r^) gene in *Halobacterium *sp. NRC-1. This replicating plasmid vector was then transformed into the respective *Halobacterium *sp. NRC-1 Δ*ura3 *strain containing the gene deletion plasmid which had been stably integrated into the specific targeted gene locus. Excisants of the gene deletion vector were selected using Foa^r ^while the replicating plasmids were maintained with mevinolin selection (Fig. 1B). Individual colony isolates were screened for the presence of wild-type or deletion alleles of the chromosomal copy of the gene of interest, in the same manner that the aforementioned non-essential gene knockout strains were screened (Figs 4B, 5B, 6B and Table 2).

Replicating plasmids containing a functional, *polD1 *(pBB*polD1*all)*, polB1 *(pBB*polB1*all)*, mcm *(pBB*mcm*all),* pri2 *(pBB*pri2*all), or *rad2 *(pBB*rad2*all) gene plus the native promoter were introduced into a *Halobacterium *sp. NRC-1 Δ*ura3 *strain containing the corresponding deletion plasmid, respectively, integrated into the chromosome. After selection for excisants using Foa^r ^and Mev^r ^selection, candidate clones were screened for wild-type or deletion alleles of either *polD1*, *polB1* (Fig 4B), *mcm, pri2* (Fig 5B) or *rad2 *(Fig 6B) genes using PCR with primers external to the genes. Since the plasmid borne genes contained only ~100 bp of 3'-flanking region and the 3'-end primers mapped > 500 bp downstream, the PCR assay was specific for the chromosomal genes. Our results showed that one or more chromosomal deletants were obtained for *polD1*, *polB1*, *mcm, pri2*, and *rad2 *genes (Figs 4B, 5B, 6B, and Table 2) only when a complementing wild-type copy was provided on a replicating plasmid. These results confirm the requirement of the five genes for cell viability using both statistical and genetic criteria. Attempts to cure selected replicating plasmid vectors in strains containing a chromosomal gene deletion by growing in media lacking mevinolin selection for many generations and screening for presence of the mevinolin resistance marker displayed that these vectors were stably maintained in the absence of exogenous selection, unequivocally displaying the essential nature of the DNA replication gene carried on the plasmid (data not shown).

## Discussion

Analysis of DNA replication components in archaeal systems has been restricted primarily to bioinformatic analysis and *in vitro *biochemical characterization. However, in our investigations, we have utilized the power of genetics in *Halobacterium *sp. NRC-1, to study DNA replication in this model Archaeon. Previously, we defined the *cis *acting elements required for chromosomal and pNRC100/200 DNA replication [8,9]. In the current study, we have examined the *in vivo *essentiality of nineteen genes for predicted components of DNA replication initiation and elongation. Ten genes are most likely required, encoding two Orc/Cdc6 origin recognition proteins, two DNA polymerases (one B and both subunits of the D family), four accessory proteins, the replicative helicase protein MCM, primase proteins Pri1/Pri2, processivity clamp protein PCNA, and Okazaki fragment maturation protein Rad2. Taken together, our results provide a better view of the likely *in vivo *requirements for DNA replication in *Halobacterium *sp. NRC-1.

Significantly, our study has targeted the largest number of genes for deletions in any archaeon to date [6,7,21-33]. For the first time, we have used statistical analysis of gene knockout frequencies and in several cases complementation analysis to critically evaluate the essentiality of genes for which deletions could not be recovered. Statistical analysis showed that the probability of recovering knockout mutants is > 99.999 % in all cases where 40 potential candidates were screened. Where no mutants were observed (*orc2*, *orc10*, *polD1*, *polD2*, *polB1*, *mcm*, *pri1*, *pri2*, *pcn*, and *rad2*), we have very strong evidence for the requirement of these genes for cell viability. In five cases tested by complementation analysis (*polD1*, *polB1*, *mcm*, *pri2*, and *rad2*), knockouts were recovered when a functional copy of the gene was present on a replicating plasmid, confirming that the genes were essential to cells and also dominant *in trans*. These results provide a genetic system for further analysis of essential DNA replication genes in *Halobacterium *sp. NRC-1.

Interestingly, we found that only two of ten *orc *genes encoded in *Halobacterium *sp. NRC-1 are essential. We had previously hypothesized that *orc7 *and likely *orc6 *would be essential for viability, based upon our previous genetic work showing the requirement of *orc7 *for autonomous replication ability of a minichromosome plasmid replicon [8]. Biochemical work performed on an Orc7 ortholog in *S. solfataricus *[15] and a chromatin immunoprecipitation study in *Pyrococcus abyssi *[13] are also consistent with the function of Orc7 proteins in chromosomal origin binding proteins in Archaea. However, we found the *orc7 *gene of *Halobacterium *sp. NRC-1 to be dispensable under standard growth conditions. Because NRC-1 contains ten *orc/cdc6 *homologs, it is possible that another gene may be functionally redundant to *orc7 *in this archaeon. In contrast, Orc7 orthologs are found in a single gene copy in most other Archaea, with the exception of *Sulfolobus *spp. which have two *orc7 *orthologs linked to two chromosomal DNA replication origins [15,16].

Most Archaea encode an *orc6 *gene ortholog in their genomes [8], but our genetic analysis shows this gene is also not essential to *Halobacterium *sp. NRC-1. *Sulfolobus *spp. Orc6 proteins have been found to bind origin DNA sequences, although in partially synchronized cultures, expression of the Orc6 ortholog appears to be in G_2 _phase cells [15]. It is possible that Orc6 orthologs act as negative regulators of DNA replication initiation, preventing re-replication by binding to origin sequences and blocking binding of replication initiation factors. Both the Orc7 and Orc6 orthologs from *Methanothermobacter thermoautotrophicus *have also been shown to interact with MCM and inhibit helicase activity, with the Orc6 ortholog being a more potent inhibitor [34,35]. It is also possible that Orc6 orthologs in Archaea act as Cdc6 does in eukaryotes, recruiting the replicative helicase complex to DNA replication origins. In *Halobacterium *sp. NRC-1, the *orc6 *gene is not essential for viability and no discernable phenotypes are observed when it is deleted, possibly as a result of functional redundancy.

Surprisingly, we found *orc10 *on the large chromosome, and *orc2 *on pNRC200 are essential. Although these genes are not found to be conserved in the genomes of non-halophilic Archaea, there are likely orthologs and paralogs found in all halophilic Archaea. Orc10 shares 50% sequence similarity to the non-essential Orc8 protein from *Halobacterium *sp. NRC-1. It also shares sequence similarity to Cdc6-3 from *N. pharaonis *and eight homologs from *H. marismortui*, including a previously unrecognized Orc/Cdc6 homolog on the pNG500 replicon (Fig. 2), and at least three homologs from *Haloferax volcanii *(data not shown). Interestingly, the *orc10 *genetic locus harbors an ISH12 element 100 bp from the *orc10 *predicted translational start codon and is also an area of the large chromosome with extrachromosomal characteristics, e.g. an increased AT% and a higher concentration of IS elements [10]. Orc2 is over 90 % identical in amino acid sequence to Orc4 and shares sequence homology with Orc5 and Orc3 from *Halobacterium *sp. NRC-1, and forms a clade with Cdc6-5 from *N. pharaonis*, four homologs from *H. marismortui*, and at least seven homologs from *H. volcanii *(data not shown). At this time we cannot strictly state that the *orc10 *and *orc2 *genes are essential for DNA replication, only that they are essential for viability of *Halobacterium *sp. NRC-1, although their homology to the other haloarchaeal, archaeal, and eukaryotic *orc*/*cdc6 *genes would strongly indicate that they are involved in some essential and thus far uniquely haloarchaeal role in DNA replication (Fig. 2). It is tempting to speculate that these two *orc *gene products play an important role in coordinating cell cycle and DNA replication of the chromosome and extrachromosomal replicons in *Halobacterium *sp. NRC-1. It is possible that they function as the origin binding proteins for the large chromosome and pNRC200, respectively, or they may be required to recruit the replicative helicase, or additional replisome components in haloarchaea. Moreover, our recent unpublished work has shown that the *orc10 *and *orc2 *genes are essential in mutants harboring multiple *orc *gene knockouts, while also indicating that some *orc *gene products are non-essential even in strains already having knockouts of other *orc *genes.

All sequenced haloarchaea to date contain at least one homolog in each of the five Orc/Cdc6 phylogenetic clades (Fig. 2). The large haloarchaeal *orc/cdc6 *gene family may therefore represent an evolutionary scenario, similar to eukaryotes, in which gene duplication events followed by functional divergence have led to evolution of heteromeric protein complexes for origin recognition. With discrimination of essential vs. non-essential *orc *genes, it will be interesting to determine if heteromeric Orc/Cdc6 complexes form in *Halobacterium *sp. NRC-1 and to identify specific functions and interactions of individual gene products.

Our results also show that two replicative-type DNA polymerases are absolutely required for *Halobacterium *sp. NRC-1. Both of the chromosomally encoded DNA polymerases, the B family *polB1 *polymerase, and the D family *polD1*/*polD2 *polymerase, are essential. From *in vitro *biochemical characteristics determined with the *Pyrococcus *B and D family DNA polymerases [36,37], it would appear that the euryarchaeal specific heterodimeric D family polymerase, PolD1/PolD2, may act at the lagging strand and the B family polymerase, PolB1 may act at the leading strand. The B family polymerase can only use DNA primers for extension, while the D family polymerase can use either RNA or DNA primers for extension, though it requires PCNA for efficient DNA synthesis [38]. However, these points are speculative and require more direct genetic and biochemical experiments to confirm. 

The non-essentiality of *polB2 *is also interesting. PolB2 contains the ten conserved polymerase and exonuclease motifs of archaeal B family DNA polymerases (data not shown), so it would appear to be a functional DNA polymerase. A PolB2 homolog is also found in the genome of the distantly related halophile, *H. marismortui*, on extrachromosomal replicon pNG600. Of interest, as well, is the fact that in both *Halobacterium *sp. NRC-1 and *H. marismortui*, the *polB2 *gene is divergently oriented with respect to an Orc5 clade member gene [17]. The function of this evolutionarily conserved genetic linkage between *polB2 *and an Orc5 clade member gene in these two haloarchaea is currently unknown, but in *Halobacterium *sp. NRC-1 both *orc4 *and *polB2 *are non-essential genes. While much *in vitro *work has been directed at determining the properties of archaeal DNA polymerases, especially since the discovery of a novel DNA polymerase family in euryarchaea [19], no *in vivo *analysis had previously been performed to determine whether these DNA polymerase family members were essential, consistent with a requirement for DNA replication.

For the other five accessory genes examined here, whose products comprise four protein complexes, the results were as expected: *mcm*, *pri1*, *pri2*, *pcn*, and *rad2 *are essential for normal growth of *Halobacterium *sp. NRC-1. The *in vitro *biochemical work done on these various gene products had indicated that it was likely that they would function in an analogous manner to their eukaryotic homologs. Though no biochemical work has been done on the haloarchaeal MCM, our genetic analysis is consistent with its predicted function as a replicative helicase. With Pri1/Pri2 (homologs of the eukaryotic p48 and p58 proteins), the archaeal complex likely acts as the DNA-dependent RNA primase for DNA replication. The finding of the essential nature of the *pri1 *and *pri2 *genes in *Halobacterium *sp. NRC-1 is consistent with their role as a replicative primase. In contrast, the function of the bacterial-type primase, DnaG, coded by most archaeal genomes, including *Halobacterium *sp. NRC-1 is unknown, although in *S. solfataricus *it has been reported to be associated with the archaeal exosome [39]. For PCNA, the function is likely to be as a DNA polymerase sliding clamp. While most Archaea possess a single gene for *pcn*, similar to eukaryotes, two crenarchaea, *S. solfataricus *and *Aeropyrum pernix*, are exceptions, with three *pcn *genes each, reminiscent of the eukaryotic 9-1-1 complex [40,41]. In *Halobacterium *sp. NRC-1, we have found that the single *pcn *gene is essential, consistent with PCNA acting as the homotrimeric DNA polymerase sliding clamp. Rad2 family flap endonucleases are important in both the processes of DNA replication, (during Okazaki fragment maturation), and repair (in nucleotide excision repair). Organisms can possess multiple homologs, although just a single flap endonuclease gene was detected in the genome of *Halobacterium *sp. NRC-1 [17]. Genetic studies in yeast indicate that *rad27*, the *rad2*/FEN1 homolog in *S. cerevisiae*, is not essential unless a recombination gene (e.g. *rad51 *or *exo1*) is also deleted [42]. In the present investigation, we have shown that the *rad2 *gene is essential for viability of *Halobacterium *sp. NRC-1. This finding is consistent with flap endonucleases being required for DNA replication via their role in Okazaki fragment maturation in this archaeon.

The results obtained in this and a previous investigation [8] are relevant to most other archaeal organisms, with the large *orc *gene family representing a unique aspect of DNA replication in haloarchaea. In our emerging model, archaeal chromosomal DNA replication origins are comprised of a large inverted repeat flanking an AT rich DNA sequence proximal to the gene encoding an origin binding protein, an *orc*/*cdc6 *gene that is an *orc7 *ortholog. These large inverted repeats likely serve as binding sequences for the origin binding protein, probably Orc7, although a multimeric ORC complex or other Orc proteins, especially the *orc2 *and *orc10 *gene products cannot be ruled out. Binding of origin recognition protein(s) would lead to local DNA helix destabilization of the intervening AT rich region allowing for recruitment of the essential *mcm *gene-coded replicative helicase complex, potentially by the *orc6 *gene product, followed by association of other replisome components, such as the essential eukaryotic-type primase (*pri1/pri2 *gene products). Once the primase lays down an RNA primer at the origin, the essential *pcn *gene product may be loaded onto the primed template and essential B (*polB1*) and D (*polD1/polD2*) family replicative DNA polymerases. The *rad2 *gene product encodes the likely flap endonuclease which helps to mature Okazaki fragments. During the replication process, the *polB1 *gene product coding the B family DNA polymerase may act as the leading strand DNA polymerase and the *polD1 *and *polD2 *gene products coding the D family DNA polymerase may act as the lagging strand DNA polymerase for processive and faithful duplication of the genome.

By utilizing a well developed in-frame gene knockout system in *Halobacterium *sp. NRC-1, we have established a foundation on which to explore further the *in vivo *roles of these DNA replication genes. With facile genetics, complete genome sequence, and established post-genomic methodologies, *Halobacterium *sp. NRC-1 provides an excellent model system to further study the characteristics of archaeal DNA replication. In addition, the gene knockout and complementation methodology used for studying DNA replication in *Halobacterium *sp. NRC-1 may be applied to the investigation of many other aspects of archaeal biology [2].

## Methods

### Materials

Restriction enzymes, calf intestinal phosphatase, T4 DNA polymerase, T4 polynucleotide kinase, and T4 DNA ligase were purchased from New England Biolabs, Beverly, MA. XL DNA Polymerase was purchased from Applied Biosystems, Branchburg, NJ. Oligonucleotides were purchased from Sigma-Genosys, The Woodlands, TX. Gel extraction kits and plasmid purification kits were purchased from Machery-Nagel, Easton, Pa. Uracil dropout formula and Nitrogen base were purchased from Sigma-Aldrich, St. Louis, MO.

### Strains and culturing

*Escherichia coli *DH5α was grown in Luria-Bertani medium supplemented with 100 μg of ampicillin/mL at 37°C. *Halobacterium *sp. NRC-1 Δ*ura3 *was cultured in CM^+ ^medium containing 4.3 M NaCl, trace metals, and 250 μg/mL of 5-Foa at 42°C [4,5]. *Halobacterium *sp. NRC-1 Δ*ura3 *containing integrated suicide plasmids were grown in HURA^+ ^medium at 42°C [7].

### Gene knockouts

To generate gene knockout suicide plasmid vectors, regions surrounding the target gene were PCR amplified from wild-type *Halobacterium *sp. NRC-1 genomic DNA (see Table 1 for pBBΔ plasmid series, oligonucleotide sequences, and number of codons remaining after deletion). PCR products were then digested with appropriate restriction enzymes and cloned into the multiple cloning site (MCS) of plasmid pBB400, which contains a wild-type copy of the *Halobacterium *sp. NRC-1 *ura3 *gene plus its native promoter [5]. Two independent suicide plasmid vector isolates for each gene were then individually transformed into *Halobacterium *sp. NRC-1 Δ*ura3 *via the PEG-EDTA methodology [4]. Transformation cultures were then plated onto HURA^+ ^solid media and grown 7–10 days at 42°C. DNA from individual colony isolates was then used as template in PCR reactions to verify suicide plasmid integration into genomic DNA. Two isolates were then plated onto CM^+ ^solid media containing 250 μg/mL of 5-Foa and grown at 42°C for 7 days. Colonies were then picked from the CM^+ ^solid media containing 250 μg/mL of 5-Foa and grown at 42°C for 7 days in liquid CM^+ ^media containing 250 μg/mL of 5-Foa. Genomic DNA was extracted from these cultures and used as template in PCR reactions to screen for knockout alleles using primers which flanked the target gene.

### Complementation

To further address the question of essential genes we developed a complementation strategy [5]. In this method, a wild-type copy of the gene of interest plus its native promoter was PCR amplified (see Table 1 for pBBall plasmid series and primers sequences) and cloned on a replicating plasmid vector, pNG168 [3,4], containing a selectable marker (*mev*^r^) and then transformed into the *Halobacterium *sp. NRC-1Δ*ura3 *strain harboring an integrated copy of the original suicide vector. Subsequent selection for suicide plasmid excision (Foa^r^) and replicating plasmid maintenance (Mev^r^), by plating on CM^+ ^solid media containing 20 μg/mL of mevinolin and 250 μg/mL of 5-Foa, results in selection of chromosomal knockouts, even if the targeted gene is essential, due to complementation *in trans *by the plasmid borne wild-type allele of the gene.

### P-value calculation

Taking the null hypothesis H_0_=*geneX *is non-essential with the probability of identifying the wild type allele P_WT _= 0.75, the probability of identifying 40 out of 40 wild-type alleles is P = 10^-5^, providing strong evidence to reject H_0_.

### Sequence analysis

Protein sequences for *Homo sapiens*, *Drosophila melanogaster*, and *Saccharomyces cerevisiae *were downloaded from KOG1514 and KOG2227 at NCBI. Protein sequences for *Halobacterium *sp. NRC-1 and *Haloarcula marismortui *were generated locally. Sequences for *Natronomonas pharaonis *and *Arabidopsis thaliana *were downloaded from NCBI. Protein sequences were aligned using CLUSTAL_X1.83 and alignments manually inspected. Quartet puzzling maximum likelihood phylogenic analysis was performed with TREEPUZZLE5.2 using the JTT amino acid substitution matrix.

## Competing interests

The authors declare that they have no competing interests.

## Authors' contributions

BRB performed research, with assistance from PD, and drafted the manuscript. SD supervised the research, including design, data analysis, and finalized the manuscript, with assistance from PD.
